# Sexy Mouth Odour? Male Oral Gland Pheromone in the Grain Beetle Parasitoid *Lariophagus distinguendus* (Förster) (Hymenoptera: Pteromalidae)

**DOI:** 10.1155/2015/216952

**Published:** 2015-10-22

**Authors:** Kerstin König, Lucy Seeger, Johannes L. M. Steidle

**Affiliations:** Institute for Zoology, Animal Ecology (220c), University of Hohenheim, 70593 Stuttgart, Germany

## Abstract

Throughout the animal kingdom, sexual pheromones are used for the attraction of mates and as courtship signals but also enable sexual isolation between species. In the parasitic wasp *Lariophagus distinguendus*, male courtship behaviour consisting of wing fanning, antennal stroking of the female antenna, and head nodding stimulates female receptivity leading to copulation. Recently *L. distinguendus* was reported to consist of two different lineages, which are sexually isolated because males fail to elicit receptivity in foreign females. It is unclear, however, which part of the courtship behaviour triggers female receptivity and therefore could be a mechanism causing sexual isolation. Here we show that in *L. distinguendus* a nonvolatile male oral pheromone is essential to release the female receptivity signal. In contrast, male wing fanning and antennal contact play a minor role. Additionally, the composition of the oral pheromone depends on the developmental host and females learn the composition upon emergence from the host substrate. These results will enable more detailed work on oral sexual pheromones to answer the question of how they are involved in the speciation process of *L. distinguendus* and other parasitoid species, for a better understanding of the huge biodiversity in this group.

## 1. Introduction

In many animals, sexual pheromones are involved in mate finding and courtship but also enable sexual isolation between species [1] mediated by pheromone divergence [2, 3]. This is also true for the hyperdiverse group of hymenopterous parasitoids [4, 5]. Here pheromones are used for long-range and short-range attraction of mating partners (e.g., [6–10]) as well as during courtship. Thereby, female derived compounds stimulate wing fanning behaviour, mounting, and specific courtship behaviours by males, which in turn induce receptivity by the females (e.g., [11–16]). Male courtship behaviours often consist in head nodding and/or antennal stroking movements of the female's antenna and it has been suggested that male pheromones are applied during this process [5]. The role of sexual pheromones in parasitoid speciation has been studied in detail with the pteromalid wasp* Nasonia vitripennis* Walker (Pteromalidae) [17, 18]. The divergence of pheromones between closely related species was addressed for* Drosophila *parasitoids [19–21].

Because many parasitoid wasps are used for biological pest control, knowledge on their biology is potentially relevant for their application. For example, the parasitoid wasp* Lariophagus distinguendus* (Förster) (Pteromalidae) is used for the biological control of stored product pests as the granary weevil* Sitophilus granarius *(L.) (Dryophthoridae: Curculionoidea), for many years [22, 23]. Its establishment as biocontrol agent was based on detailed studies on its biology (e.g., [24–28]). Recently, we discovered that* L. distinguendus* consists of two genetically distinct lineages, which most likely represent two species. One is specialised on drugstore beetles (*Stegobium paniceum *(L.), Anobiidae) in pantries and has a low fecundity on granary weevils, whereas the other attacks granary weevils and is mostly found in grain stores [29]. Hence it is not advisable to use the first species for biocontrol of granary weevils. A strong indication for the species status of the two lineages consists in the fact that they are sexually isolated and females do not accept males from the other lineage as mating partners (König et al., in prep.). This suggests a communication breakdown between sexes during courtship. The courtship behaviour of* L. distinguendus* consists of male wing fanning, stimulated by the female cuticular hydrocarbon profile (CHC profile), followed by mounting and antennal stroking of the female antennae by the males. Subsequently, females signal receptivity by lowering their head and open their genital orifice leading to copulation [12, 30–33]. It is unclear if this receptivity signal is stimulated by vibrations due to the wing fanning behaviour of the males [16], by pheromones transferred via the male antennae as suggested in other hymenopterous parasitoids (e.g., [34]), or by a male oral gland pheromone as in the related species* N. vitripennis *[35]. Therefore, the reason for the communication breakdown causing sexual isolation between the two* L. distinguendus* lineages remains to be clarified.

To answer this question, we studied the origin (antennae or mouthparts) and the nature (tactile or chemical) of the male courtship signal of* L. distinguendus*, which induces receptivity in females. We analysed the role of antennae and mouthparts of the males during mating behaviour via video recordings, performed an experiment on the role of antennal contact during courtship, examined courtship success of males with sealed mouthparts, and studied the volatility of a putative pheromone. Finally, because it is known that the composition of sexual pheromones can be influenced by the feeding substrate of an insect [36–38], we addressed the question if development on the two different hosts of the* L. distinguendus* lineages, drugstore beetles and granary weevils, might have caused sexual isolation.

## 2. Material and Methods

### 2.1. Insects

For all experiments we used* L. distinguendus* wasps from the SLOgw strain [29]. Wasps were reared in Petri dishes (9 cm diameter) on 40 g wheat grain (cultivar: Batis; Saaten-Union GmbH, Hannover, Germany) infested by either drugstore beetles or granary weevils. Insect cultures were kept under constant conditions of 26°C and 45% r.h. and 16 L : 8 D photoperiod. For host rearing, 1 g of adult unsexed drugstore beetles or 2.7 g of adult unsexed granary weevils was placed on 40 g wheat grains moistened with 1 mL H_2_O. After six weeks wasps were placed on the grains infested by drugstore beetle larvae or after three weeks on the grains infested by granary weevil larvae. Developmental time of* L. distinguendus* was 17–21 days. After wasps emerged out of the grain and before having contact to possible mating partners, males and females were kept separately in small Petri dishes (diameter 5.5 mm). For all experiments 2-day-old wasps were used.

### 2.2. Position of Antennae and Male's Mouthparts during Courtship

To analyse the role of antennae and mouthparts of the males during mating, we observed and videotaped 20 matings. Virgin males and females were placed in an arena consisting of a glass Petri dish (diameter 30 mm) closed with a glass plate (30 mm × 30 mm) and the mating behaviour was video recorded using a Digital Handheld Microscope (Bresser Meade Instruments Europe) fixed on a metal lab support stand. Videos were recorded with 7.5 fps and 1280 × 1024 pixels for a maximum time of 20 minutes or up to copulation behaviour. The camera operates with integrated software for video recording. Magnification was adjusted between 20x and 200x. During the subsequent analysis of the videos we focused on the position and movement of male and female antennae and on male's mouthparts.

### 2.3. Role of Antennal Contact

To examine if antennal contact is required for releasing the female's receptivity signal, we studied mating success of 20 couples with cross-ablated antennae, that is, after removing the right antenna of the male and the left antenna of the female (*n* = 10) and vice versa (*n* = 10) using a scalpel. Wasps were anaesthetised before the ablation by cold temperature (−23°C for 1.5 min). This procedure has no effect on wasp behaviour (data not shown). After removing the antenna, wasps were allowed to recover for 30 minutes. Experiments were conducted in the same arena as described above and the behaviours (wing fanning, antennal stroking, receptivity signal, and copulation) were registered by direct observation for a maximum of 20 min using a stereomicroscope (Zeiss Stemi SV11).

### 2.4. Mouthparts as Source of a Putative Pheromone

To test if a pheromone is released from the male mouthparts, mating experiments were performed with males with sealed mouthparts. Males were collected and mated with virgin females in order to check their ability to release the female receptivity signal. Subsequently, males were anaesthetised by cold temperature (−23°C for 1.5 min) and mouthparts were sealed with solvent-free superglue (UHU easy geruchsfrei, UHU GmbH & Co. KG, Bühl, Germany). To ensure that sealing of mouthparts with glue did not affect the activity of the males, they were kept in a Petri dish for 3 h before being used in the experiments. Males which were inactive during this period were discarded. In the experiments, single males were placed into a mating arena as described above together with one virgin female. Mating behaviour [12] consisting of “wing fanning,” “antennal stroking/head nodding,” “receptivity signal,” and “copulation” was registered for a maximum of 20 minutes using a stereomicroscope. After the first test, each male was retested with a second virgin female for another 20 minutes. When no copulation occurred, females were paired with a second, untreated male. The experiment was performed with 20 males with sealed mouthparts (test) and 20 untreated males (control). To exclude that the presence of glue on the males did affect the experiments, males were treated with a drop of glue on their thorax. Mating experiments were conducted with these males as described above with the exception that only one female per treated male was tested.

### 2.5. Volatility of the Putative Pheromone

To study the volatility of the putative male pheromone, an experiment from van den Assem et al. [39] with* N. vitripennis* was repeated with some modifications. In a first experiment, two couples of* L. distinguendus *were placed in one small glass vial each (Supelco, 2 mL Clear Vial, Screw Top). In one couple, the male's mouthparts were sealed as described above; in the other couple the male was untreated. By using a gastight syringe, air was collected from the headspace close to the antennae of the couple with the untreated male during antennal stroking. This air was injected next into the antennae of the couple with the treated male, again during antennal stroking. Then, the occurrence of copulation was registered using a stereomicroscope. The experiment was repeated 20 times.

In a second experiment, the two couples were placed in direct neighbourhood to each other during mating behaviour. Each female was fixed with superglue (s.a.) at the tip of one dissecting needle (Supplementary Figure S1 in Supplementary Material available online at http://dx.doi.org/10.1155/2015/216952). A wheat grain was placed in the middle under the females to enable males mounting the females. To enable that needles are moveable in all directions, they were pinned in a block of polystyrene fixed in a metal sphere, which was placed on a metal ring. Males were released onto the females. As soon as males started antennal stroking, females were brought into close distance from each other (<2 mm) but without having direct contact by moving the needles. Again, the occurrence of copulation was registered using a stereomicroscope. The experiment was repeated 15 times.

### 2.6. Effect of Developmental Host and Experience on Sexual Isolation

To study if development on the two different hosts might cause sexual isolation, mating experiments were performed with males and females which were reared on granary weevils or for one generation on drugstore beetles. Single couples of virgin males and females from the same or from different hosts were placed in an arena as described above and the behaviours (wing fanning, antennal stroking, receptivity signal, and copulation) were registered using the software package THE OBSERVER 9.0 (Noldus) for a maximum of 20 min or until copulation occurred. To examine the role of experience, the experiment was performed twice. First, we tested naïve wasps without emergence experience, which were dissected out of the grain as pupa and kept in an Eppendorf tube until emergence (*n* = 50). Second we tested experienced wasps, which were collected directly after the emergence out of the grain (*n* = 50). The contact period of these wasps with grain after emergence was <1 min. The occurrence of the different behaviours was compared and statistically analysed separately for naïve and experienced wasps using the 4 × 2 *χ* ^2^-test for an overall comparison, followed by Bonferroni corrected 2 × 2 *χ* ^2^-test for single comparisons.

## 3. Results

### 3.1. Position of Antennae and Male's Mouthparts during Courtship

The analysis of videos from 20 matings showed that during courtship the female antennae were stretched forward in a V-shape. Males were sitting on the female with their front legs placed on the female's head, performed wing fanning, and moved their antennae in skewed circles starting from above their heads downwards. The right antenna was moving clockwise and the left one counterclockwise (Supplementary Video 1). During circling, the male antennae often touched the middle of the female's antennae, stroking forward to their tip and rising up again in the air, starting a new circle. Thereby, the female's antennae were bended out of the V-shape into a rather parallel position (Supplementary Video 2). This brought them into the vicinity of the male's mouthparts, which were moved forward and backward along the female's antennae by head nodding. The mandibles were always in close vicinity (Supplementary Video 2), but not always in direct contact with the female's antennae, while contact of the mandibular or labial palps cannot be excluded (Supplementary Video 3).

### 3.2. Role of Antennal Contact

In these experiments one antenna was ablated in each male and each female in opposite positions to prevent direct contact of antennae during courtship behaviour. Nevertheless, all males showed normal wing fanning and antennal stroking behaviour. Likewise, all females gave the receptivity signal and successful copulation was observed in all 20 couples tested.

### 3.3. Mouthparts as Source of a Putative Pheromone

**Figure 1:**
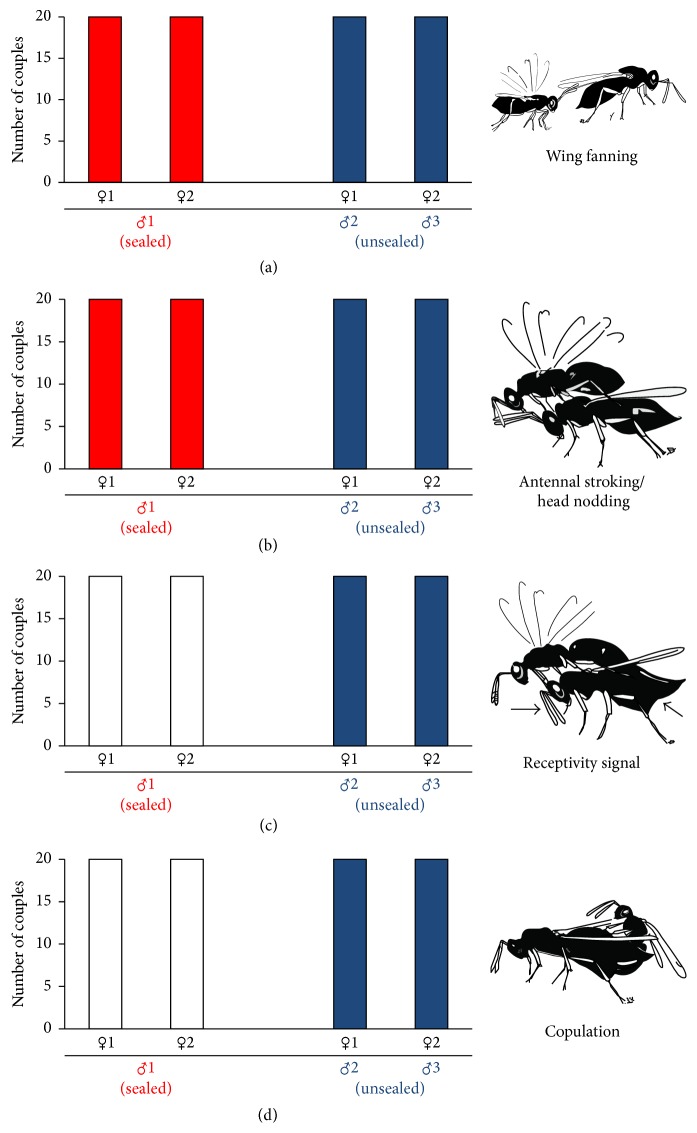
Occurrence of the different mating behaviours ((a) wing fanning, (b) antennal stroking/head nodding, (c) receptivity signal of female, and (d) copulation) in couples with a male in which the mouthparts had been sealed with superglue (red) and in couples with an unsealed male (blue). White bars indicate that the specific behaviour did not occur.

When mouthparts of males were sealed with superglue, males performed wing fanning and antennal stroking behaviour, but females did not show the receptivity signal and therefore no copulation occurred (Figure 1). However, when tested females were paired afterwards with untreated males, they all mated readily (Figure 1). Likewise, copulation occurred in all 20 control experiments, in which males were treated with a drop of glue on their thorax.

### 3.4. Volatility of Putative Pheromone

When the headspace above couples with an untreated male was transferred using a gastight syringe to couples with males with sealed mouthparts, no copulation could be observed (*n* = 20). Likewise, when two couples were brought into close vicinity, the couple with the unsealed male mated normally, while the couple with the sealed male showed no copulation (*n* = 15).

### 3.5. Effect of Developmental Host and Experience on Sexual Isolation

**Figure 2:**
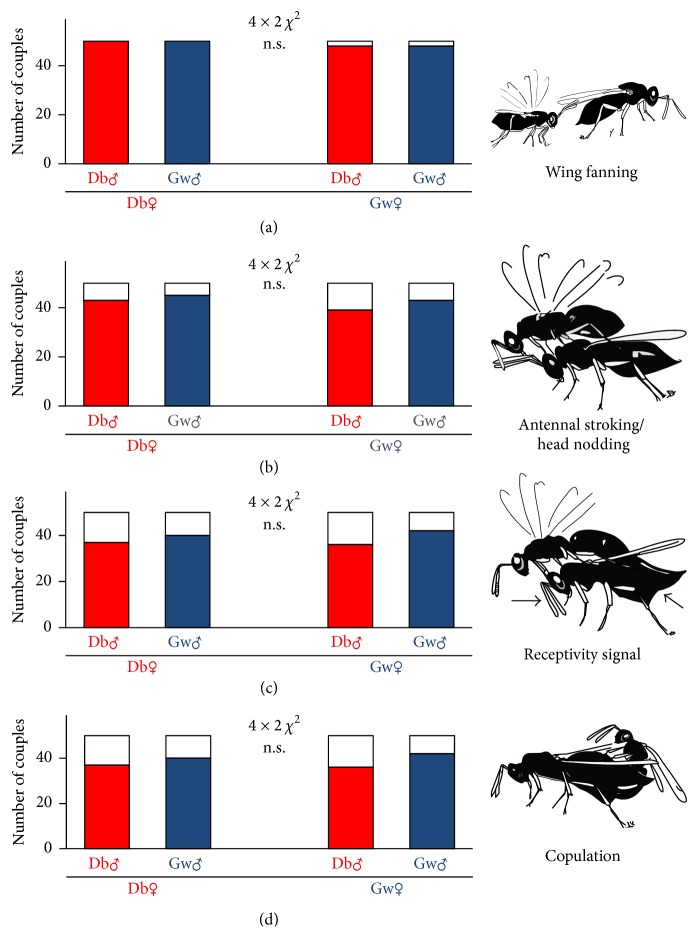
Occurrence of the different mating behaviours ((a) wing fanning, (b) antennal stroking/head nodding, (c) receptivity signal of female, and (d) copulation) in naïve wasps. Red bars are couples with a male that developed on drugstore beetles (Db); blue bars refer to couples with males that developed on granary weevils (Gw). White parts of bars indicate couples, which did not show the specific behaviour. n.s.: not significant (overall comparisons using 4 × 2 *χ* ^2^-test).

**Figure 3:**
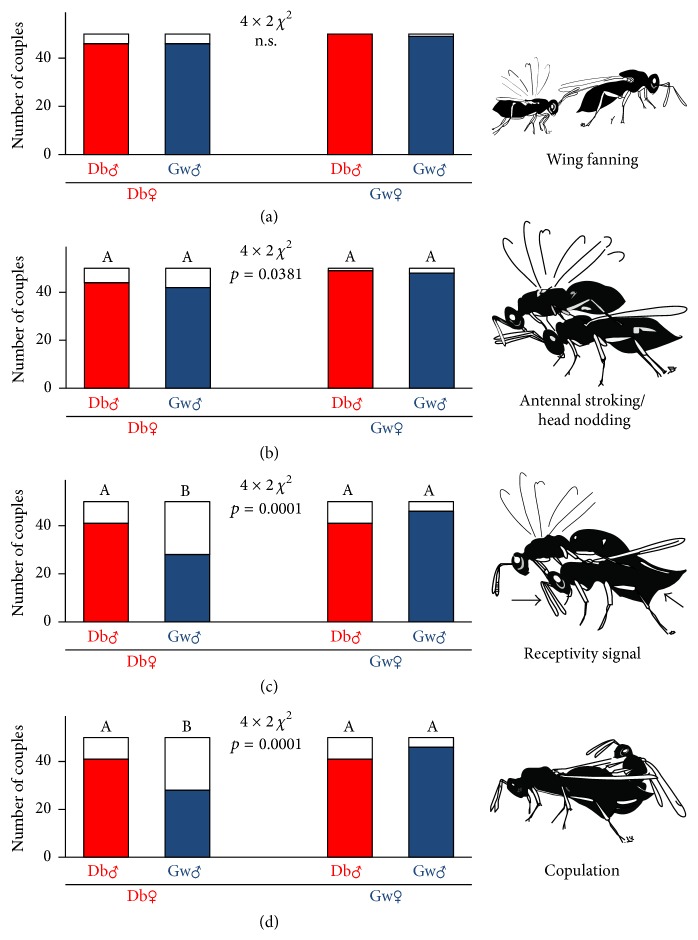
Occurrence of the different mating behaviours of wasps with emergence experience from the host substrate ((a) wing fanning, (b) antennal stroking/head nodding, (c) receptivity signal of female, and (d) copulation). Red bars are couples with a male that developed on drugstore beetles (Db); blue bars refer to couples with males that developed on granary weevils (Gw). White parts of bars indicate couples, which did not show the specific behaviour. *p* values refer to overall comparisons using 4 × 2 *χ* ^2^-test. n.s.: not significant. Bars with different lowercase letters are significantly different at *p* < 0.05 (Bonferroni corrected 2 × 2 *χ* ^2^-test).

When wasps were naïve, that is, dissected out of the grains in which they developed, no significant differences were found between the experimental groups for all behaviours, including receptivity signal and copulation (Figure 2). When wasps hatched normally out of the grain, no overall differences were found for wing fanning but for antennal stroking, receptivity signal, and copulation. Single comparisons followed by Bonferroni correction revealed no differences between experimental groups for antennal stroking, but for receptivity signal and copulation. In couples consisting of females that had developed in drugstore beetles and males from granary weevils significantly less receptivity signals and copulations were observed (Figure 3).

## 4. Discussion

Despite the fact that mating behaviour in* L. distinguendus* has been studied by several authors before [12, 32, 33, 40] it was still unclear what triggers receptivity in the female. Our analysis of video recordings from matings revealed that during courtship males perform wing fanning and touch the antennae of females with their own antennae and with their mouthparts during head nodding. This is in line with the observations of the former studies [12, 32, 33, 40]. Therefore, based on these earlier studies and our video recordings, receptivity in females could be induced by males through mechanical stimuli due to wing fanning (hypothesis 1) or antennal contact (2), volatile pheromones from antennae (3) or mouthparts (4), or nonvolatile pheromones from antennae (5) or mouthparts (6).

Our experiments with wasps, in which antennal contact between males and females was prevented by cross-ablation of antennae, revealed normal matings in all couples tested. Thus antennal contact between mating partners is not necessary to induce copulation. This excludes the hypotheses that the female's receptivity signal is stimulated by mechanical cues via antennal contact or by a male contact pheromone, which is transferred via the male antennae onto the antennae of the females (hypotheses 2 and 5).

In mating experiments with males having mouthparts sealed with superglue no copulations could be observed, despite the fact that males performed normal wing fanning and antennal stroking. This strongly supports the hypothesis that the male's mouthparts are essential for stimulating the female's receptivity by releasing a volatile or nonvolatile pheromone (hypothesis 4 or 6) and falsifies the idea that pheromones from the antennae are involved (hypotheses 3 and 5). Because the closely related* N. vitripennis* uses a male oral pheromone as well [35], this seems to be a general trait for Pteromalidae. Although the role of wing fanning has not been explicitly studied, our results also demonstrate that wing fanning alone is not sufficient (hypothesis 1). In agreement with Benelli et al. [16] we assume that it might play a role as additional signal indicating male quality to enable mate choice decisions by the females.

To analyse the volatility of the male pheromone, we tried to stimulate mating behaviour in a couple with a sealed male by exposing it to putative volatile compounds transferred from a normal couple via a syringe and to a mating couple within 2 mm distance. In both cases, no receptivity signal by the female could be stimulated. These results contradict hypotheses 3 and 4 from above and let us assume that the mandibular male pheromone is nonvolatile, acting only at contact or at very close distance (hypothesis 6). Again, this agrees with* N. vitripennis*, where the male oral pheromone has been demonstrated to be nonvolatile and is transferred via contact directly onto the female's antennae [5, 35].

The experiments on the effect of the developmental host on the male pheromone showed that females which developed on drugstore beetles accepted males from granary weevils significantly less often as mating partners than males from their own developmental host. Due to the low relevant *p* values, ranging from *p* < 0.000 to *p* < 0.0049, and the use of the Bonferroni correction we strongly assume that this result is not based on an alpha-error. It points to the fact that the developmental host influences the composition of the pheromone from the male's mouthparts. This agrees with findings from the CHCs profile of* L. distinguendus* [41] but also with other insects, where pheromone composition has been reported to depend on the feeding substrate (e.g., [36–38, 42]).

Interestingly, the reduced acceptance of males from the other developmental host was only observed in experienced females, which emerged normally out of their grains, but not with naïve females, which were dissected from their grains. Thus, experience gained during emergence out of the grain must have affected the female's behaviour. This learning process could consist in direct learning of chemical host cues by imprinting upon emergence, which has been demonstrated recently in* L. distinguendus* [29].

The rejection of males was only observed in encounters of females from drugstore beetles and males from granary weevils, but not vice versa. We hypothesise that pheromones from males developing in granary weevils contain all compounds present in pheromones of males from drugstore beetles, whereas the latter contain additional compounds. Therefore, females developing in drugstore beetles reject males from granary weevils because they miss important compounds in the male pheromones, which are synthetised only when wasps develop in drugstore beetles. Alternatively, females might be able to learn at emergence only cues from drugstore beetle, but not from granary weevils. Thus, only the former influenced acceptance or rejection of males.

## 5. Conclusions

Our study strongly supports the hypothesis that the release of a nonvolatile oral pheromone by the males is essential to induce female receptivity in* L. distinguendus*. We found no support for alternative hypotheses, as mechanical stimuli due to wing fanning or antennal contact, volatile or nonvolatile pheromones from the antennae, and volatile pheromones from the mouthparts. Based on our video recordings we assume that the primary function of the antennal stroking is to move the female's antennae into the vicinity of the male mouthparts where the pheromone is applied. A putative source for this oral pheromone could be the mandibular glands, which are described by several authors for* L. distinguendus* [40, 43] and* N. vitripennis* [44].

Interestingly, the composition of this pheromone seems to be host dependent and is learned by the female during development, possibly during emergence from the host. This enables sexual isolation by development on different hosts within one generation, a phenomenon that has been also described for other insects (e.g., [36–38]). It remains to be studied if this mechanism has played a significant role for the sexual isolation of the two lineages of* L. distinguendus.* Further studies need to address the chemical identification of the pheromone. This will considerably help to answer the question of how pheromones were involved in this speciation process. In addition, our study enables more detailed work on oral sexual pheromones and their role in speciation in parasitoids, for a better understanding of the huge biodiversity in this group.

## Supplementary Material

Figure S1: Setup for the experiment to test the volatility of a putative pheromone when the two couples were placed in direct neighbourhood to each other.
Video S1: Circular movement of male's antennae.
Video S2: Bending female antennae out of the V-shape by males antennal stroking behaviour.
Video S3: Contact between male's mandibular or labial palps and females antennae.
