# Supplementary material for: Sexy Mouth Odour? Male Oral Gland Pheromone in the Grain Beetle Parasitoid Lariophagus distinguendus (Förster) (Hymenoptera: Pteromalidae)
Source: Biomed Res Int. 2015 Oct 22;2015:216952. doi: 10.1155/2015/216952 (PMC4633539; doi:10.1155/2015/216952)
Supplement: Supplementary file 1 — Figure S1: Setup for the experiment to test the volatility of a putative pheromone when the two couples were placed in direct neighbourhood to each other. Video S1: Circular movement of male's antennae. Video S2: Bending female antennae out of the V-shape by males antennal stroking behaviour. Video S3: Contact between male's mandibular or labial palps and females antennae. [file 216952.f1.zip › 216952.f1/Supplementary Figure S1.docx]

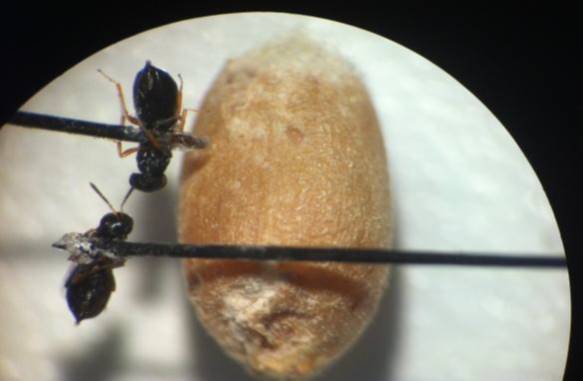


*Figure S1:* Setup for the experiment to test the volatility of a male pheromone. Two females were placed in direct neighbourhood to each other (distance < 2 mm) during mating behaviour. Each female was fixed with superglue at the tip of one dissecting needle. A wheat grain was placed in the middle under the females to enable males mounting the females.
